# The option of transosseous distal suture placement during minimally invasive Achilles tendon repair for high-risk patients can improve outcomes, however does not prevent re-rupture

**DOI:** 10.1186/s12891-024-07630-8

**Published:** 2024-08-01

**Authors:** Michael R. Carmont, Katarina Nilsson-Helander, Malin Carling

**Affiliations:** 1 Department of Trauma & Orthopaedic Surgery, Shrewsbury & Telford Hospital NHS Trust, Shropshire, UK; 2 University of Keele, Staffordshire, UK; 3 Department of Orthopaedic Surgery, Mölndal Hospital University of Gothenburg, Gothenburg, Sweden; 4 The Department of Orthopaedics, Institute of Clinical Sciences at Sahlgrenska Academy, Gothenburg University, Gothenburg, Sweden

**Keywords:** Achilles repair, Transosseous, Transtendinous

## Abstract

**Purpose:**

Achilles tendon ruptures (ATRs) close to the insertion, in high-level athletes, and in patients at high risk of re-rupture, may be better suited to operative repair. Minimally Invasive Repair (MIR) of the Achilles tendon has excellent outcome and low complication rates. Traditionally MIR has showed lower repair strength, failing due to suture pull-out from the distal tendon stump. The aim of this study was to describe the outcome of ATR patients who received transosseous distal suture placement using a standard technique as a reference.

**Methods:**

Following ATR, patients were evaluated for pre-injury activity level, body weight, location of the tear and size of the distal Achilles tendon stump. Patients considered to be at high-risk of re-rupture: Tegner level ≥ 8, body weight ≥ 105Kg and distal ATR, received transosseous (TO) distal suture placement (*n* = 20) rather than the usual transtendinous (TT) technique (*n* = 55). Patient reported outcome measures and functional evaluation was performed at 12 months following repair.

**Results:**

At 12 months follow up both methods resulted in good median (IQR) Achilles tendon Total Rupture Score TO 83.8 (74-88.3) vs. TT 90 (79–94), low increased relative Achilles Tendon Resting Angle TO -3.5˚ (3.6) vs. TT -3.5˚ (3.3) and mean (SD) Single leg Heel-Rise Height Index TO 88.2% (9.9) vs. TT 85.6% (9.9) (n.s.). There were 4 re-ruptures in the high-risk group and 2 in the group receiving TT distal suture placement. All but one of these were traumatic in nature. The mode of failure following TO distal suture placement was proximal suture pull out.

**Conclusions:**

To distal suture placement during minimally-invasive Achilles tendon repair for higher-risk patients can lead to results equivalent to those in lower-risk patients treated with a standard TT MIR technique, except for the re-rupture rate which remained higher. There may be factors that have greater influence on outcome other than suture placement following ATR.

## Introduction

Prospective randomised controlled studies have shown that operative treatment optimises the recovery of ankle plantar flexion strength [1], reduces tendon elongation [2] and re-rupture rate [3] following Achilles Tendon Rupture (ATR). Minimally-invasive repairs of ATR have been shown to give good outcome and low complication rates compared with open repairs, in randomised controlled trials and meta-analyses [4, 5], and now may be considered to be the gold standard technique of a mass end-to-end repair of the ruptured Achilles tendon.

The aim of orthopaedic sports medicine research is to improve overall outcome for patients and minimise the burden of the complications of management. From 2009 minimally-invasive repair (MIR) of the Achilles tendon has been performed in our unit, consisting of a modified Bunnell suture in the proximal tendon end and a Kessler suture in the distal stump [6]. Biomechanical studies show that this suture configuration tends to fail by suture pull out from the distal tendon end [7, 8]. Patients in our unit who sustained re-ruptures of the Achilles tendon were examined and evaluated to determine factors that occurred in patients sustaining re-rupture. Ruptures at the distal Achilles insertion and ruptures in high-level athletes or other patients at high risk of re-rupture, may be better suited to MIR with sutures placed into the calcaneum rather than the distal suture stump. Biljsma and van der Werken [9] describe a technique that incorporates a Bunnell suture proximally and distally the suture passed through a transverse drill hole in the calcaneum. Using absorbable polydioxanone suture, (PDS) this technique produces excellent outcome with little complications and low re-rupture rates [10–12]. Amlang M et al. [13] also described transcalcaneal suture placement for distal ATR. More recent techniques use a polyester non-absorbable locked suture in the proximal stump which is anchored directly into the calcaneum using polyethylene ether ketone anchors (PEEK) [14–17].

**Fig. 1:**
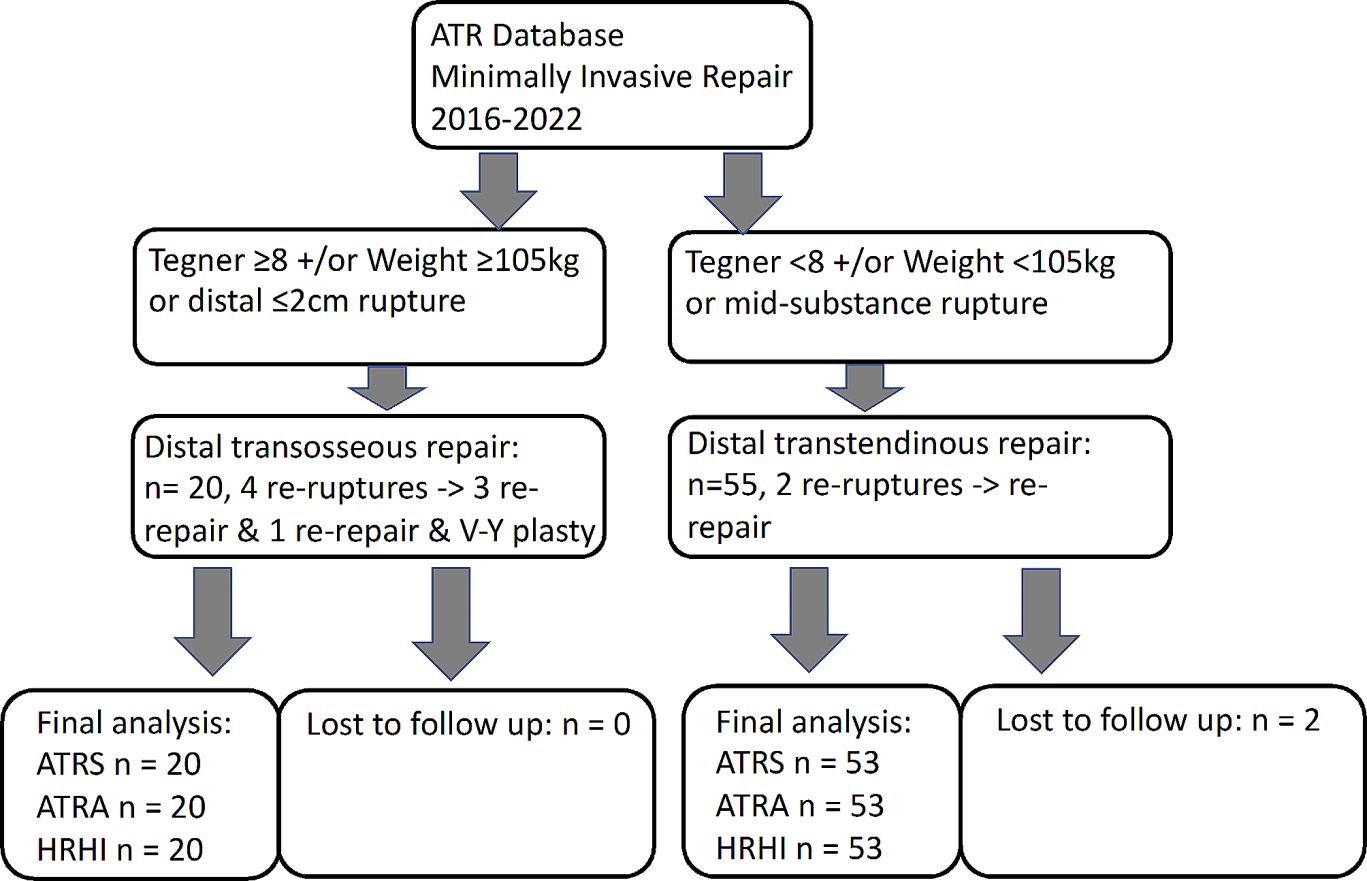
Flow diagram of Achilles Tendon Rupture (ATR) patients receiving TO and TT repair. Patients suffering re-ruptures were included in follow up

In biomechanical studies [7, 8], literature from the Netherlands [10–12] and the unit’s experience [18–20], patients with distal ruptures, heavy patients and competitive sports participants were considered to be at high-risk of re-rupture. These patients underwent a modified repair using non-absorbable suture through a transosseous (TO) calcaneal distal placement (Fig. 1) followed by early active rehabilitation. The aim of this study was to describe the outcome of ATR patients who received TO distal suture placement using a standard technique as a reference.

## Patients and methods

From 2016 all patients with ATR were prospectively evaluated at Princess Royal Hospital Telford. The Research and Innovation Committee of Shrewsbury & Telford Hospital NHS Trust have approved the study and confirmed that this study is Service Evaluation and that Ethical Application is not required. All patients gave their informed consent for participation in the study and publication of their anonymous data. All patients demonstrated the triad of a palpable gap, the absence of plantar flexion with the calf squeeze test and an increased relative Achilles Tendon Resting Angle [19] confirming the diagnosis of Achilles tendon rupture [21]. Patients were counselled of the outcomes, the rates and burden of complications of both operative and non-operative management. Patients therefore made an informed choice of treatment. Patients received minimally invasive surgery, and were included if they received surgery within 14 days from the injury. Patients were followed up until 12 months following injury. The outcome of these patients has been subsequently reported [18–20] and patients who sustained re-rupture have been evaluated for the patient characteristics and the mode of failure of the repair at re-repair or reconstruction (Table 1). Repairs that failed did so consistently at the original rupture site at the mid-substance of the tendon due to suture pull out from the distal stump are site in all patients, rather than knot failure. Additional factors were that the patients were heavy or had high pre-injury Tegner [22] level of activity. Therefore, a TO rather than TT suture placement was therefore used in the presence of one of the following criteria: palpable tendon gap was within 2 cm of the tendon insertion, patient weight ≥ 105 kg, had a high pre-injury Tegner scale ≥ 8 (Fig. 1). The standard technique was used if the palpable tendon gap was more proximal than 2 cm from the tendon insertion [6]. Patients with musculotendinous ruptures and those presenting at more than 2 weeks following injury were excluded.

**Table 1:** Demographic characteristics and mechanism of re-rupture following MIR prior to 2016

Age	Gender	Time following repair/weeks	Weight/Kg	Tegner	Mechanism of repair failure	Mode of failure
51	M	9	145	5	Spontaneous	Distal pull out
47	F	8	105	4	Slip shower	Distal pull out
43	F	5	73	8	Spontaneous	Distal pull out
45	M	4	106	8	Slip bath	Distal pull out
49	F	12	115	6	Spontaneous	Distal pull out

### Transosseous technique

**Fig. 2:**
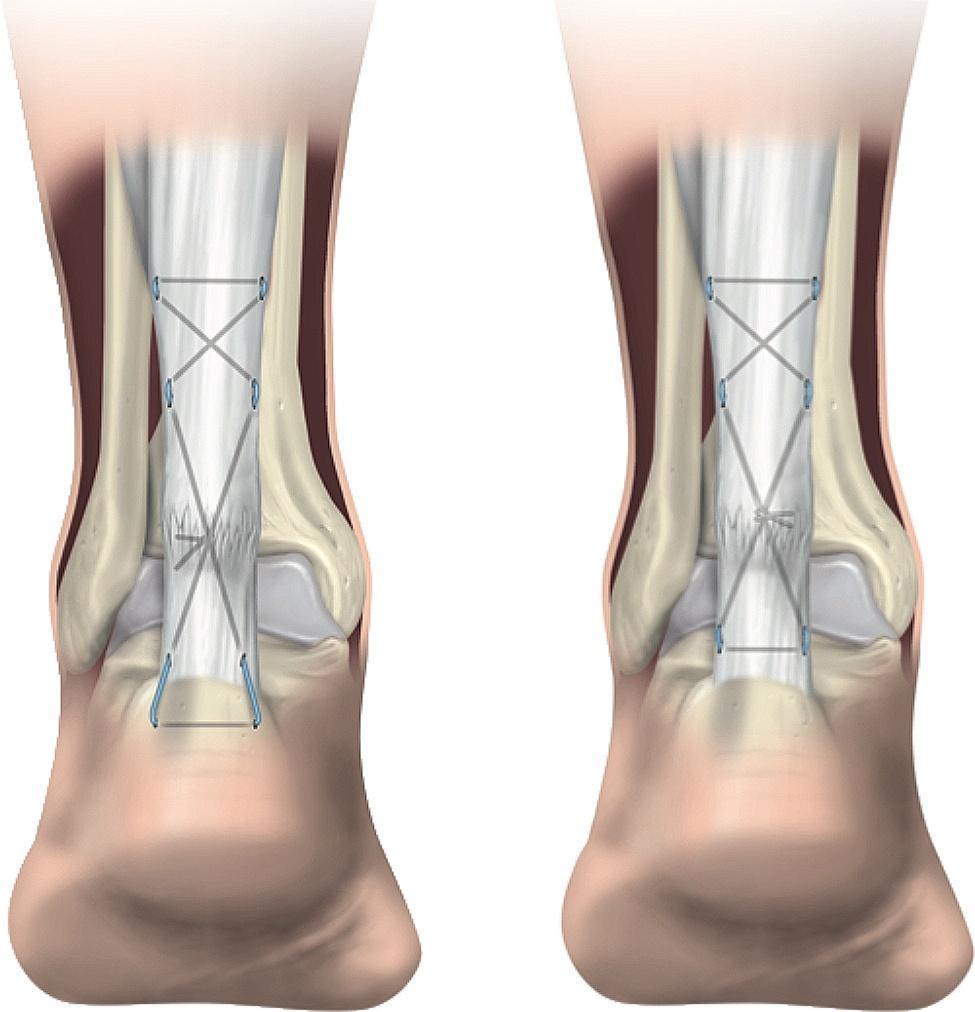
Illustration of the Transosseous (**2a** Left) and Transtendinous (**2b** Right) repair techniques

Patients underwent a repair of the Achilles tendon rupture using a minimally invasive technique modified [19] from a previous percutaneous technique [6]. A 2 cm medial longitudinal incision just proximal to the rupture site, allowed visualization of the tendon ends apposition following repair. A second 2 cm longitudinal mid lateral incision at 8–10 cm from the insertion was made to visualize and protect the sural nerve. Repairs were performed using a No. 2 non-absorbable polytetrafluoroethylene coated polyester suture (Fiberwire^®^, Arthrex, Naples, Fl) using a Bunnell configuration proximally. The distal incisions were positioned either side of the Achilles insertion. A 2.5 mm drill was then used to make a TO tunnel across the calcaneum at the level of the Achilles insertion. A straight 5 cm needle was then passed through this hole so that 3 strands of No. 2 non-absorbable polytetrafluoroethylene coated polyester suture (Fiberwire^®^, Arthrex, Naples, Fl) were passed through the suture loop, and the needle withdrawn so that the sutures pass across the calcaneum. In turn either ends of the sutures were passed subcutaneously into the distal stump and tendon before emerging through the medial incision (Fig. 2a). These intra-tendinous sutures were tied with the ankle and foot held in maximal plantar flexion or reduced ATRA to the non-affected side. Knots were buried within the tendon substance, or placed anteriorly to the tendon and the fascia cruris was closed using absorbable detensioning absorbable sutures.

### Transtendinous technique

Patients underwent a repair of the Achilles tendon rupture using a minimally invasive technique [19] modified from a previous percutaneous technique [6]. The proximal suture being a modified Bunnell technique and distally a transtendinous Kessler suture. With the ankle held passively in full plantar flexion the proximal and were tied in the manner described above (Fig. 2b).

Patients receiving the TO technique received general anaesthesia and patients with the TT technique offered the choice between general or local anaesthesia. All patients received 30mls of 0.5% Bupivucaine with 1:200,000 adrenaline local anaesthetic field infiltration. Other aspects of the procedure were the same for both groups. Pre-operative flucloxacillin 1 g and six weeks of low molecular weight heparin (Tinzaparin 4500iu once daily (Leo Pharma, Berkshire, UK)) were administered. Once the local anaesthetic was worn off and protective pain sensation restored, patients were encouraged to weight-bear on their metatarsal heads as tolerated by pain. The patient used a protective synthetic equinus split cast for 2 weeks and crutches. After this period, the split cast was removed only for early active movement exercises consisting of plantar flexion, inversion and eversion contractions of 10s duration performed for 10 repetitions, 3 times per day. Dorsiflexion was not permitted until 6 weeks. Weight-bearing continued using the anterior protective shell alone, secured in place using elasticated velcro straps. After the six-week time point the functional brace was discontinued and patients were given a 1.5 cm in-shoe heel wedge. Departmental physiotherapy was commenced at the 6-week time point consisting of gait retraining and strengthening with double heel rises progressing to single heel rises. Stretching and plyometric exercises were avoided until the 3-month time point. No formal restrictions were given with regards to return to play, however, most patients held off returning to play until they felt comfortable to do so typically 6–9 months following rupture.

### Re-repair technique

Re-repair was performed using an incision over the rupture site scar. This was extended proximally and distally until the mode of failure could be determined, the existing suture removed and the tendon ends mobilised. If end-to-end apposition could be achieved the repair was repeated using the original technique of a modified Bunnell suture proximally and either a TO or TT Kessler suture for distal placement using 6 strands of No. 2 Fiberwire. Re-repair was performed using the original repair technique provided tendon end apposition could be achieved. Thus, TO re-rupture repair was performed using a TO technique and a TT re-rupture repair using a TT technique. A proximal V-Y plasty was performed to lengthen the proximal stump and permit end-to-end apposition in the one case where this was not possible at the re-rupture site. The larger incision enabled the repair to be augmented by a No. 2 polyglactin running circumferential suture. This technique is the same as that performed for Acute-on-Chronic repairs [23]. The post-operative regime was the same as per the original repair.

### Follow-up evaluation

Patients were reviewed at 6 weeks, 3, 6, 9 and 12 months after surgery by the same examiner (MC) with the advantage of avoiding interobserver error. Patient outcomes were included based on intention to treat, meaning that those patients who sustained re-rupture and re-repair were included in the final follow up data. Patient reported outcome and function were evaluated using the Achilles tendon Total Rupture Score (ATRS) [24], Tegner activity scale [22] and Physical Activity Scale (PAS) [25]. The pre-injury level of physical activity/patient perception of performance (PPP) was determined at the 3-month time point [20]. At each follow-up the patients were asked, “Do you think you have reached the same level of physical activity and performance as before your injury?” They were asked to categorize their response as either: not yet attained, the same or greater/improved.

Patients were examined for palpable tendon gaps and tendon continuity using a calf squeeze test. The ATRA [26] was measured following rupture, surgery, at 6 weeks and at all subsequent evaluations. The ATRA assessment has been independently validated and shown to be reliable, reproducible and responsive [26, 27]. A maximal single leg heel-rise height (HRH) [28] and calf circumference was compared with the non-affected side at 3, 6, 9, and 12 months respectively. The calf circumference was measured using a tape measure at 15 cm below the medial knee joint line [26]. Heel-Rise Repetition (HRR) was assessed by counting the number of single-leg heel rises performed by the patient to exhaustion at the 12 months point. Finger-tip contact with the wall was permitted for balance. Limb Symmetry Index (LSI) for the Heel-Rise Height Index (HRHI) and Heel-Rise Repetition Index (HRRI) were determined as the maximal height of a single heel rise on the injured side/the maximal height of a single heel rise on the uninjured side x 100. The HRRI was calculated as the number of repetitions performed on the repaired side/number of repetitions on the uninjured side x 100.

All patients provided written consent for data collection and this research was considered to be service evaluation according to the Health Research Authority and consequently ethical approval was not required.

### Statistical evaluation

All data were analysed using Statistical Package for Social Sciences version 28 (IBM Corp, Armonk, NY). Patients with TO ATR were compared with the cohort of patients with TT AT repair.

Descriptive statistics were reported using mean and standard deviation (SD) and median (25th -75th percentile Inter-Quartile Range (IQR)) values. Outcomes were compared according to intention to treat i.e. patients who underwent re-repair surgery were included in final outcome. An ATRS of 80 points was considered to be good [29]. Due to the numbers of patients involved non-parametric statistics were performed Mann-Whitney U test. Categorical analysis was performed using Cross Tabs and Fischer’s Exact test. Repeated measures of the ATRA over time were compared using Two Way ANOVA. Statistical significance was taken to be < 0.05. All patients who met the inclusion criteria were included in the study. Therefore, no sample size calculation was performed as all patients were included.

## Results

Prior to April 2016, the rate of re-rupture was 2.8% (5/180) patients. The use of distal suture TO placement was introduced for patients considered to be of high risk. Seventy-five patients were subsequently repaired following ATR with either TO or TT distal suture placement (Fig. 1). The TO group had no loss to follow up. Two patients who had received TT distal suture placement, were lost to follow up prior to the 9 months evaluation point. The demographics of the two groups is shown in Table 2.

**Table 2:** Patient demographics

	Transosseous	Transtendinous	*p* value
Number	20	55	N.A.
Age/Years	43 (11.6)	43.8 (12.2)	0.805
Gender/ F: M	4:16	11:44	1
Side/ L: R	12:8	30:25	0.794
Tegner Pre-injury	6.3 (1.8)	6.8 (1.4)	0.235
Weight/kg	100.1 (15)	84.1 (13.3)	< 0.001
Body Mass Index	31.6 (4.8)	27.2 (3.7)	< 0.001

Data listed as Mean (Standard Deviation), F = female, M = male, L = left, R = right. N.A. = Not applicable

The frequency of complications and the patient’s subsequent management is shown in Table 3. Eleven patients (20%) reported altered sensation from the sural nerve distribution following injury and prior to operative repair 20% (4) TO 12.7% (7) TT. Re-rupture was categorized into spontaneous, during active push off, or traumatic due to a fall and forcible landing on the affected limb. All re-ruptures occurred at the mid-substance, the site of the original rupture. In the transosseous group the mechanism of failure was from suture pull out of the proximal tendon. There were no suture or knot failures. All re-ruptures failed due to a single traumatic event when excess load was placed on the foot to avoid falling. In the transtendinous group both re-ruptures occurred at the original rupture site, from distal suture pull out and due to a traumatic event in one case and a spontaneous rupture during walking in the other.

**Table 3:** Complications and the patient’s subsequent management

	Transosseous (*n* = 20) %(n)	Transtendinous (*n* = 55) %(n)
Re-rupture	20 (4) 4 Traumatic, 2w, 3w, 8w -> 3 Re-repair, 1 12w Re-repair & VY plasty	3.6 (2) 1 Traumatic, 4w -> Re-repair 1 Spontaneous, 12w -> Re-repair
Infection	0	1.8 (1) -> Oral antibiotics
Sural nerve injury	5 (1)	1.8 (1)
Adhesion	5 (1) -> Endoscopic debridement & distal suture removal	1.8 (1)
Chronic Regional Pain Syndrome	0	1.8 (1)
Deep Venous Thrombosis	5 (1)	5.5 (3)

The number followed by w is the number of weeks following primary repair the re-rupture occurred. -> = went onto

**Table 4:** Outcome parameters at 12 months following repair based upon intention to treat

	Overall	Transosseous	Transtendinous	*p* value	95% CI
ATRS/Points					
Median(IQR)	87(74–94)	83.8 (74-88.3)	90 (79–94)	0.977	-2.5, 13.7
Mean(SD)	81(16.5)	78.3(16)	83.8 (17)		
Tegner	5.33(1.7)	5.5(2.2)	5.3(1.5)	0.77	4.9, 5.7
Change in Tegner	-1.3(1.6)	-0.8(1.9)	-1.5(1.3)	0.15	-1.7, -0.95
PAS	4.5(0.9)	4.2(2.1)	4.7(3.5)	0.08	4.3, 4.7
ATRA/˚	-3.5(3.5)	-3.5(3.6)	-3.5(3.3)	0.977	-1.8, 1.9
Difference Calf Thickness Mean(SD)/cm	-1.4(1.4)	-1.3(1.5)	-1.5(1.3)	0.451	-0.99, 0.45
HRHI Mean(SD)/%	86.9(12)	88.2(9.9)	85.6(14.2)	0.39	-10.4, 4.11
HRRI Mean(SD)/%	81.6(17)	79.8(16.3)	83.3(17.6)	0.516	-7.1, 14.0

CI = Confidence Interval, IQR = Inter Quartile Range, SD = Standard Deviation, % = percent, SD = Standard Deviation, PAS = Physical Activity Scale HRHI = Heel-Rise Height Index, HRHI = Heel-Rise Repetition Index

**Fig. 3:**
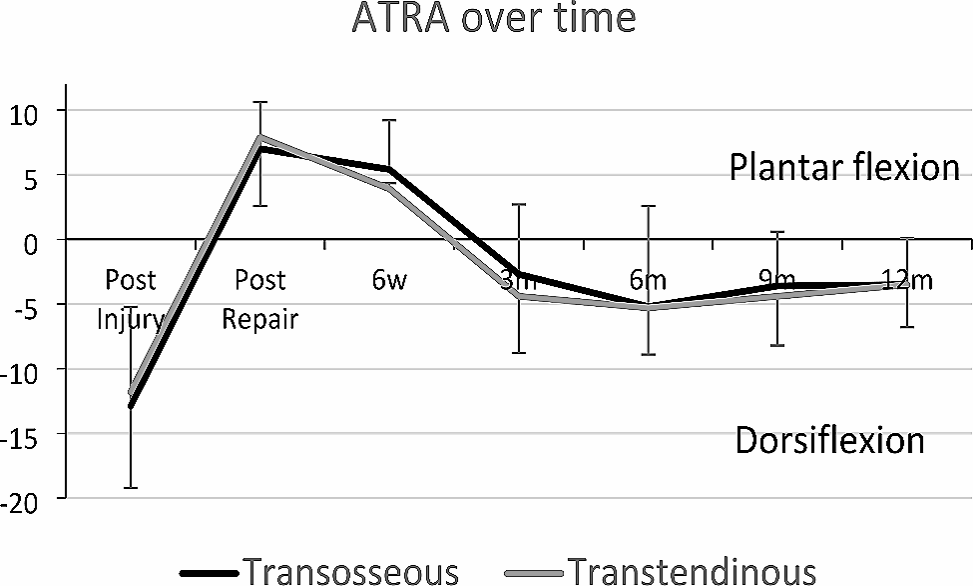
The ATRA over time for Transosseous and Tendinous distal fixation

The progress of the ATRA over time is illustrated in Fig. 3. There was no significant difference in ATRA between surgical techniques. The patient reported and functional outcomes are reported in Table 4. Only one patient was unable to perform a Single Heel-Rise by 12 months following repair. This patient received a TT repair and subsequently received a lumbar sympathetic blockade for chronic regional pain to the limb.

In terms of Return to Sports and activity performed in the TO group there was no median change in Tegner scale at one year however only 4/15 patients reported the same level of sports performance. In the TT group the median change in Tegner level was a reduction of one level. There was no difference in PAS with a mean score of 4.2 and 4.7 in the TO and TT groups respectively. 40% of patients reported at least the same perceived sports performance, including 2 patients who reported better performance than prior to ATR.

## Discussion

The most important finding of this study was that the option of distal suture fixation with minimally invasive Achilles tendon repair for high-risk patients can improve outcomes, however this technique did not prevent re-rupture. Patients receiving TO and TT repair had good outcome with very little weakness to their calf function and 87% of HRHI. The Relative ATRA was within 4 degrees suggesting little elongation using either method. Most patients had returned to their pre-injury sports, however there was a reduction in Tegner level of 1.5 at one year following injury and the majority of patients felt their performance was lower than pre-injury.

Using the TT technique with an absorbable suture [6], Maffulli et al. [30] reported an isometric plantar flexion strength of 92.2% of the non-injured side together with a relative ATRA of -3.7˚. The outcomes of a proximal Bunnell suture and a distal Bunnell suture with TO passage using a Fiberwire suture has, to our knowledge, been first reported by Nguyen et al. [31] in 2022. Patients reported an ATRS of 91 points, all were able to perform a single heel-rise at one year and there were no cases of elongation. These results show a consistently high functional outcome using this MIR technique from different units. A heel-rise height index of 80% was reported following stable open repair with early functional rehabilitation in the RCT in Olsson et als. series [32]. HRHI values of 84.5% and 86.9% were reported for minimally-invasive Dresden repair and open repair in Myhrvold et als. [3] recent randomised controlled trial.

The outcomes of the TT suture group appear to be better than the 6-strand group published in 2017 median ATRS of 93, a relative ATRA of -4.8 ˚ and a HRHI of 81% [20]. The option of a distal TO placement enables patients who were at risk of tendon elongation, as well as re-rupture, to be removed from the TT group, improving this groups outcome.

The TO method was used for patients that were considered to have a high risk of re-rupture such as those who are very competitive, heavy patients or those who have very distal Achilles ruptures close, within 2 cm of the Achilles insertion. The patients in the TO group are very heterogenous according by virtue of the inclusion criteria. Patients with high Tegner level tend to have a low weight and vice versa. This is reflected by the lack of significant differences in the group demographics.

The numbers of patients who sustained re-ruptures in the TT group was small however the numbers of re-rupture were double in the high-risk group managed using a TO. The patients selected as being high-risk of re-rupture were at greater risk of re-rupture. It is important to note that all re-ruptures in the TO group failed due to suture pull through from the proximal tendon stump and none due to distal TO pull through or knot failure. This is a change in the location of the tendon-suture interface failure within the repair construct. In the proximal tendon a Bunnell suture, which has been shown to be a strong suture configuration was used, however the Bunnell suture is not a locked suture. The use of a locked suture in the coronal plane using the Percutaneous Achilles Repair System (PARS) (Arthrex, Naples, FL), was not included in Yammine et al’s meta-analysis [33], and has been shown to give a strong ultimate tensile strength [34] and may offer stronger pull-out strength than the Bunnell suture. Other factors include the mechanism and timing of re-rupture. In the TO group all re-ruptures were traumatic in nature occurring due to a fall following a trip or slip when the patient put in excess of full body weight on the repaired tendon. The anterior shell did not supply enough support holding the ankle in plantar flexion in these circumstances and the unit’s current practice is to use an alternative method of protection for post-operative care. In Maffulli et als. [30] series using slowed down rehabilitation, a walker boot with wedges used for 12 weeks post-operatively. The outcomes are similar with ATRS 80 points, Relative ATRA − 4.5˚ and an isometric plantar flexion strength difference of 47.9 N/m.

Approximately half of patients with the TO technique complained of temporary minor discomfort, and had mild tenderness and swelling, at site of the calcaneal tunnel, however in most cases this settled by the six-month point. One patient required endoscopic debridement to remove the distal portion of the suture and retrocalcaneal bursitis. Discomfort in the area of a TO tunnel has also been reported by Jennings et al. [35] when using polyester tape and in the series from the Netherlands in which absorbable polydioxanone suture was used [10–12]. The PARS technique may avoid this by using a knotless technique anchoring sutures directly into the Achilles insertion.

This study is an attempt to modify a surgical technique, apply it to specific patients and evaluate outcome using the standard technique as a reference. Strengths include the low rates of loss to follow up. Only two patients did not attend follow up beyond the 6-months evaluation and the outcome of patients following re-rupture in the final follow up were included. The same repair technique was used following re-rupture.

Limitations of this study include the small number of patients and the heterogeneity of the TO group. The relative infrequency of distal ATR means that there is little outcome data on this patient group and that further research on techniques for managing this injury is needed [36]. The patient factors which were associated with high risk were based on the observations of the characteristics of the small number of patients who sustained re-ruptures in our unit. Greater numbers of re-rupture patients (*n* = 48) were identified from a much larger group of Health Board patients (*n* = 783) in Scotland. Male patients, typically aged < 45 years and traditional immobilisation techniques were found to be risk factors however Body Mass Index was shown to be non-significant [37]. Whilst it is important to minimise the rate and burden of complications following injury management, it is also important to balance this with optimising the outcome and return to sport and activity of all patients following ATR.

The analysis of improving the management of patients sustaining a re-rupture after receiving MI or Pc repair is a challenge mainly due to the small numbers of patients who re-rupture following this method of surgical repair. A prospective randomised controlled study may give more definite conclusions. However, to gain adequate numbers of patients to enable significant difference to be shown, such a study would likely need to be multicentre PRCT, or Registry based potentially introducing heterogeneity due to variation in the re-repair techniques used.

TO distal suture placement during minimally-invasive Achilles tendon repair for higher-risk patients can lead to results equivalent to those in lower-risk patients treated with a standard TT MIR technique, except for the re-rupture rate which remained higher. There may be factors such as post injury brace selection and duration of use, that have greater influence on outcome other than suture placement following ATR.

## Data Availability

The datasets used and/or analysed during the current study are available from the corresponding author upon reasonable request.
